# Image Based Data Mining Using Per-voxel Cox Regression

**DOI:** 10.3389/fonc.2020.01178

**Published:** 2020-07-21

**Authors:** Andrew Green, Eliana Vasquez Osorio, Marianne C. Aznar, Alan McWilliam, Marcel van Herk

**Affiliations:** ^1^The University of Manchester, Radiotherapy Related Research, Manchester, United Kingdom; ^2^Department of Radiotherapy Related Research, The Christie NHS Foundation Trust, Manchester, United Kingdom; ^3^Nuffield Department of Population Health, University of Oxford, Oxford, United Kingdom; ^4^NIHR Manchester Biomedical Research Centre, Central Manchester University Hospitals NHS Foundation Trust, Manchester Academic Health Science Centre, Manchester, United Kingdom

**Keywords:** radiation oncology, NSCLC, image based data mining, outcomes, chemoradiotherapy

## Abstract

Image Based Data Mining (IBDM) is a novel analysis technique allowing the interrogation of large amounts of routine radiotherapy data. Using this technique, unexpected correlations have been identified between dose close to the prostate and biochemical relapse, and between dose to the base of the heart and survival in lung cancer. However, most analyses to date have considered only dose when identifying a region of interest, with confounding variables accounted for *post-hoc*, most often using a multivariate Cox regression. In this work, we introduce a novel method to account for confounding variables directly in the analysis, by performing a Cox regression in every voxel of the dose distribution, and apply it in the analysis of a large cohort of lung cancer patients. Our method produces three-dimensional maps of hazard for clinical variables, accounting for dose at each spatial location in the patient. Results confirm that a region of interest exists in the base of the heart where those patients with poor performance status (PS), PS > 1, have a stronger adverse reaction to incidental dose, but that the effect changes when considering other clinical variables, with patient age becoming dominant. Analyses such as this will help shape future clinical trials in which hypotheses generated by the analysis will be tested.

## 1. Introduction

Radiotherapy (RT) is a commonly used treatment for cancer in which radiation is used to destroy tumor cells. The most common RT treatment is delivered using a beam of high energy radiation generated outside the patient (External Beam Radiotherapy, or EBRT) and directed into the patient toward the tumor. Modern RT planning techniques allow highly conformal, curative doses to be delivered in the tumor while sparing surrounding healthy tissues.

Since EBRT delivers dose from outside the patient, dose to normal tissue close to the tumor is unavoidable. It is well-known that dose in normal tissue leads to treatment side effects, and limits are set on the incidental dose deposited in normal tissue. However, the determination of dose limits is not obvious, and there is still uncertainty over which organs or sub-regions of organs are most important. Most dose analyses are done using Dose Volume Histograms (DVH), in which a cumulative dose-volume histogram is calculated for a given structure (1). DVHs require delineations, therefore finding dose sensitive regions relies on having delineated the correct anatomy before analysis, limiting analyses based on the assumptions made before analysis commences. Also, in DVH-based analyses it is impossible to identify subregions within structures, since the use of a DVH collapses all 3D dose distribution information into 1 dimension.

A persistent complication in all data mining is the fact that disease can interact with factors unrelated to treatment. For example, the lifestyle of a patient can have a profound impact on their ability to tolerate RT; in lung cancer, patients who smoke already have impaired lung or cardiac function and may suffer more, worse side effects from treatment (2). The general health of a patient prior to any treatment can also have a profound impact on their ability to tolerate treatment. Pre-existing conditions are referred to as comorbidities, and may include any condition relevant to a patients' ability to tolerate treatment. In particular, recent evidence shows that cardiac comorbidities should play a role in determining dose limits (3). Patients having pre-existing cardiac comorbidity are significantly more likely to develop radiation induced cardiac (4, 5) and further lung damage as a result of RT treatment. Importantly, it has also been shown (6) that the size of a patient's tumor is highly predictive of survival, and therefore its effect on survival cannot be ignored. A common way to include the effects of lifestyle and other factors such as tumor size is to stratify the patient population during analysis. However, this leads to many small cohorts in which there may not be enough statistical power to find a dose response relationship and is considered bad practice (7). The way such effects are typically handled in DVH based analyses is through the use of a multivariate Cox regression considering all variables found to be important in multiple univariate regressions. However, as with all DVH based analyses, this approach is limited by the prior assumptions made about which structures drive toxicity.

An interesting way to identify dose-sensitive regions is the method proposed by Witte et al. (8) in which the dose is correlated on a per-voxel basis with outcome in a large cohort of patients (typically 200–1,000). This approach has been named image based data mining (IBDM). In IBDM, the entire dose distribution is used without the need for delineations, thereby reducing the number of prior assumptions made and allowing the identification of dose-sensitive regions outside of delineated anatomy, or subregions within it. By comparing the dose in each voxel for each group of a binary classification, it is possible to identify regions in which dose is correlated with outcome—either survival or toxicity. IBDM has been applied in several areas where more traditional DVH based analyses would have been impossible. For example in prostate cancer, where dose differences in a region outside the prostate were associated with biochemical recurrence 4 years post treatment (9), and a link between excess dose and rectal toxicity in prostate patients (10). More recently, a sub-volume within the heart was identified as being dose-sensitive (11), with excess dose in this region being associated with poorer overall survival at 12 months post radiotherapy. IBDM has also been applied to the detection of a region related to radiation induced lung damage (12). At present the most advanced IBDM technique uses continuous outcome variables to produce a Spearman rank-correlation map between dose and outcome; using this method a region associated with radiation-induced trismus was found in patients treated for head and neck cancer (13). Clearly, clinical and lifestyle factors will affect outcome and must be accounted for in some way; for example, Yahya et al. (14) use a pixelwise logistic regression in their analysis of the relationship between bladder surface dose and urinary dysfunction. Similarly, Monti et al. (15) use a generalized linear model to account for the impact of age in their analysis of radiation induced lung damage. However, most applications of IBDM so far do not account for known clinical and lifestyle factors that affect outcome, with all correction for these factors being done *post-hoc*. In contrast to both Witte et al. (8), Chen et al. (9), and McWilliam et al. (11) in which *post-hoc* multivariate analyses were performed either for individual voxels or in IBDM identified regions, in this work we present the first application of a per-voxel survival analysis in a large radiotherapy cohort used to identify dose-sensitive regions. The advantage of this approach work is that the shape and extension of the region of interest is directly extracted after modulation by all variables. Using this strategy, avoids the risk of spurious results being found using IBDM, which may disappear when accounting for clinical factors that affect outcome. Ideally, IBDM should be performed in such a way that it can account for covariates and avoid this potential issue.

Recently, ten Kate et al. (16) used a per-voxel Cox regression technique to account for variability in time-to-event for the onset of dementia from mild cognitive impairment (MCI), finding a pattern of decreased gray matter volume beyond the hippocampus, as observed in MR imaging, to be predictive of time to progression to dementia in patients with MCI. Similarly, Sörensen et al. (17) use a per-voxel Cox regression technique to identify brain regions in FDG PET imaging in which reduced metabolism was associated with a shorter time to conversion from MCI to Alzheimer's dementia. To date, no time-to-event type analysis has been done per-voxel linking radiotherapy dose with overall survival. In this work we extend our previously developed IBDM methodology to incorporate non-dose factors without the need to stratify patients, and therefore lose statistical power. Our proposed method to incorporate confounding variables in the IBDM stage is the Cox-IBDM method, in which a Cox regression is performed considering relevant clinical factors as well as the planned radiotherapy dose in each voxel. Cox-IBDM allows the production of a hazard ratio map that can highlight changes in hazard across a patient's anatomy for variables when dose is considered. Permutation testing is used to assess significance, with dose being permuted relative to all other clinical variables.

We apply the Cox-IBDM method to non-small cell lung cancer (NSCLC) patients and demonstrate its value, comparing to the conventional approach used by our group and others.

## 2. Methods and Materials

A cohort of *N* = 1, 101 Non Small-cell Lung Cancer (NSCLC) patients treated with routine curative intent (55Gy in 20 fractions) between 2010 and 2013 at a single academic cancer center was collected without selection, including CT imaging used in treatment planning, and the planned radiotherapy dose distribution. Treatments were a mixture of 3D conformal RT (3DCRT) and Intensity Modulated RT (IMRT). Additional clinical information, including gender, age, tumor size, tumor stage, and performance status was also collected for each patient. Patients were labeled based on their survival 12 months post-radiotherapy; there were a total of 884 events in this cohort, with a median follow-up of 16 months (range 3–36 months) and median survival 12 months (range 1–36 months); no patients were lost to follow-up before 12 months. Our retrospective analysis of routine radiotherapy data was approved by the local institutional information governance and ethics committee (The Christie NHS Foundation Trust and Caldicott Committee). All data was anonymized, and the work carried out according to a protocol approved by the Caldicott committee.

Patient CT images were non-rigidly registered to an arbitrarily chosen reference patient CT using the Nifty Registration package [NifytReg, UCL (18)]. CT slice resolutions were approximately 1 mm in-plane (range 0.9–1.3 mm) with on average 3 mm slice thickness (range 3–5 mm). The registration was performed using intensities, with delineations used only to validate the accuracy of registration later, since not all patients had suitable delineations. The cost function used in the registration was normalized mutual information, and a penalty was added on the bending energy to regularize the registration. NifytyReg uses a B-spline registration algorithm; previous studies have shown that regions extracted with either B-spline registration or Demons based registration are largely equivalent (15). Dose distributions were registered by applying the deformation vector field derived in the registration of the CT images. Dose distributions were calculated at between 3 and 5 mm resolution and, following registration were resampled to the resolution of the reference CT. Using a subset of 386 patients for whom the heart had been delineated, the registration inaccuracy was estimated by measuring the deviation in the center of mass, and distance to agreement of heart segmentations mapped from the 386 patients and the heart contour in the reference patient. Registration was found to be accurate to within 3.9 mm left-right, 4.9 mm anterior-posterior, and 7.4 mm cranial-caudal. To take account of these uncertainties, dose distributions were blurred using a Gaussian kernel before use in data mining. To accelerate IBDM, the dose distributions were down-sampled by a factor of three in-plane, and a factor of two in the cranial-caudal direction, making voxels 7 × 7 × 6 mm. A mask is used to exclude regions outside the reference patient anatomy.

All IBDM was performed using a toolkit developed in-house. The toolkit includes implementations of the Student *T*-test used in binary IBDM (11), and an implementation of the per-voxel Cox regression technique discussed here. To explore changes in the extracted region of interest when stratifying on clinical variables, patients were split based on their performance status (abbreviated as PS); in the first case, we split into sub-cohorts with the same PS (i.e., 0, 1, 2, 3) and a Student *T*-test was performed using the survival labels defined as 0 (no event) if the patient was alive 12 months after radiotherapy and 1 (event) if the patient dies within 12 months after radiotherapy. No patients were lost to follow up <12 months post-radiotherapy. The Student *T*-test produces both negative and positive values for t, which imply different effects of excess radiation dose; negative t implies a detrimental effect of excess radiation, while positive t implies a protective/curative effect. For this reason, the t-test used is one-sided, and we assess positive and negative t for significance separately. Performance status was scored according to the ECOG performance scoring criteria, in which PS 0 corresponds to a patient who is fully active and whose disease has not impacted their daily life. PS 1 indicates a patient who is restricted somewhat, but still able to perform light work; PS 2 indicates a patient is capable of self care, and manages more than half of their waking hours out of a chair/bed. PS 3 indicates a patient capable of only limited self care, who is in a chair/bed more than half of their waking hours. PS 4 indicates a patient who is completely disabled, is bed bound and cannot perform any self-care (19). The majority of lung cancer patients present with PS 1 or 2, and some present with PS 0 or 3; very few have PS 4. Permutation testing, originally developed for neuroimaging (16, 17, 20) and applied in similar investigations in RT (9, 11, 13), was again applied here to test for statistical significance. In this method, outcome labels are permuted and the test statistic re-calculated in order to approximate the distribution of the statistic under the null hypothesis. In neuroimaging, permutation testing has been done by treating every voxel independently, thereby reducing the number of permutations needed to a handful. However, in radiotherapy voxel-to-voxel correlations exist and are strong, making this approach unsuitable; instead, we summarize the entire statistical map with a single value. In the case of analyses using the *t*-test, we randomize the survival labels and re-calculate the t-map; in the case of the Cox per voxel analysis, we randomize the event label and re-calculate the beta map. These maps are then summarized using the most extreme voxel in the map, in both cases, positive and negative values are treated separately. By performing this randomization and summarization many times, we are able to build an empirical distribution of the test statistic (t or beta) under the null hypothesis; this is then used to define iso-t or iso-beta levels corresponding to a given level of significance. The procedure as described here is one of the strongest corrections for the multiple comparison problem (21). In this work, the statistic tested is the β coefficient in a Cox proportional hazards model; the distribution of most extreme (positive and negative) β is used to set a threshold on the value of β required for significance.

### 2.1. An Illustration of the Pitfalls of Binary IBDM

As an example of how splitting a cohort and performing repeated binary IBDM to take account of clinical variables may lead to incorrect or unreliable results, we show an analysis of the cohort used in this work in which we perform binary IBDM on cohorts produced by splitting on the PS variable, both directly and in a dichotomized analysis.

Binary IBDM, using a Student t-test per voxel, was performed in four lung cancer radiotherapy cohorts produced by splitting the patients based on their performance status; from an original cohort of 1,101 [as used in (11)], 122 missing performance status records reduced the available cohort to 979, and a single patient with PS 4 was excluded from all binary analysis leaving 978 in the analysis. Of the 978 patients in the analysis, 140 were PS 0, 438 PS1, 339 PS2, and 61 PS3; a separate per-voxel *t-*test was performed in each of these four sub-groups as a simple way to account for the influence of PS on overall survival. Patients were labeled according to their survival at twelve months post-RT, and a per-voxel *t*-test performed on the dose distribution. Significant regions are highlighted by taking a contour at the 95th percentile of the t distribution approximated by permutation testing.

While it is possible to split a cohort based on PS, this leads to small sample sizes and fewer events in each group which limits the power of the analysis. In addition, performance status is not a precise measure of patient health, and includes a considerable uncertainty due to clinician judgment, and patient responses (22). Therefore, we considered it unwise to base analysis on the actual value for PS recorded in the clinical notes. However, it is reasonable to expect a PS 0/1 patient to be in better condition than a PS 2/3 patient, therefore a better approach may be to dichotomize PS, and separate the analysis of those patients having PS ≤ 1 (*N* = 578) and those with PS > 1 (*N* = 400) and perform analysis in these two groups; this is a tradeoff between the moderate loss of information inherent in going from the full PS distribution to a dichotomized version, and the additional robustness offered by a high/low PS classification. Dichotomizing in this way brings an additional benefit in the current analysis: the groups are much better balanced than when using the full PS dynamics. The result of this analysis is shown in Figure 2. We have use dichotomized PS to split our cohort for two separate binary analyses, and use the dichotomized PS measure in the Cox regression.

Splitting a cohort in this way and running separate IBDM in each sub-cohort accounts for differences between covariates in each sub-cohort, but dilutes the power of the analysis and may mean important insights are missed. In addition to PS, other clinical variables such as tumor size and age must be controlled for in any analysis. If a cohort were split up-front, each sub-cohort would be very small, making it almost impossible to generate any testable clinical hypothesis from the data; this approach is therefore doomed to failure as the cohort size dwindles with more covariates being controlled for.

### 2.2. The Per-voxel Cox Regression

To avoid splitting the cohort, we propose the use of our novel method: a Cox regression in each voxel of the dose distribution, taking account of the clinical variables of interest. By performing an analysis that inherently includes clinical variables that are relevant, we are able to control for differences between patient subgroups, while maintaining the statistical power of a large overall cohort and the geometrical power of a voxel-wise analysis. The per-voxel Cox regression uses a standard Newton–Raphson iterative method to produce a maximum likelihood estimate of the Cox model coefficients when taking into account relevant clinical variables in addition to the dose in each voxel; by excluding clinical variables, a univariate Cox regression on dose alone can be performed, however this result is not shown here. Cox model coefficients are readily converted into hazard ratio simply by taking the exponent of each voxel in the resulting image.

The product of the Cox regression tool is a multi-channel image in which each channel contains the hazard ratio for a given clinical variable. These hazard ratios vary spatially because of the spatial variation in the dose, and this spatial variation may imply dose-response relationships. To assess statistical significance in the per-voxel Cox regression, survival time, status, and all clinical variables are permuted with respect to the dose distributions, but not each other. This means that a given survival time remains associated with the same status and clinical variables, but each permutation involves a different dose distribution. In the per-voxel Cox regression, we first included low/high performance status as a categorical variable alongside the dose in each voxel; this analysis was performed on a cohort of 979 patients, including the one PS 4 patient excluded from the binary analysis. Performance status was included as a binary variable primarily to address the inaccuracy of performance status classification, but also to reduce the number of channels in the output hazard ratio map to a manageable number (4). As PS is a categorical variable with four levels, the inclusion of it in the Cox regression would lead to three hazard ratio (HR) channels associated with it, as the HR for each PS will be calculated with PS 0 as reference. By dichotomizing the PS variable as we have, we reduce the number of channels to 1 (PS > 1 with PS ≤ 1 as reference). Inclusion of PS as an ordinal variable (i.e., treating it as if continuous) was avoided due to the required implicit assumption that increases in PS are proportional; this is unlikely to be true, especially when considering the inherent uncertainty and qualitative nature of PS grading. All other variables considered in this regression are treated as continuous variables, and therefore have hazard ratios per unit increase in their respective covariate (e.g., Gy^-1^ for the dose HR).

Missing age data further reduced the cohort available for this analysis from 979 patients to 939. The result of this analysis is shown in Figure 4. The cube root of tumor size was preferred over raw tumor size values because the distribution of tumor volumes was skewed, and the cube root suppresses the effect of a small number of large tumors. Tumor volume now enters our models as effective tumor dimension in cm.

### 2.3. The Effect of GTV Dose

It may be argued that dose to the GTV is curative, whereas dose to the normal tissue is not, meaning that dose in the GTV should be treated differently to dose elsewhere. To date, we are not aware of any IBDM analysis in which dose to the GTV is treated differently to dose elsewhere, however it may have an impact.

To investigate the effects of how GTV dose is treated, we re-analyse the data but exclude voxels in the GTV from analysis. In binary IBDM, this equates to having a variable number of patients used to calculate the mean and standard deviation in each voxel. For the per-voxel Cox analysis, we treat voxels in the GTV as missing data. This leads to a variable number of patients being used for the regression across anatomy, as we only perform regressions using patients for whom all data is available, a so-called Complete Case Analysis (CCA). The CCA procedure results in at most 6% of patients being excluded in any given voxel.

We will demonstrate results in which dose to the GTV has been excluded, and compare them to the currently accepted methods in which dose to the GTV is included.

## 3. Results

### 3.1. The Pitfalls of Binary IBDM

**Figure 1:**
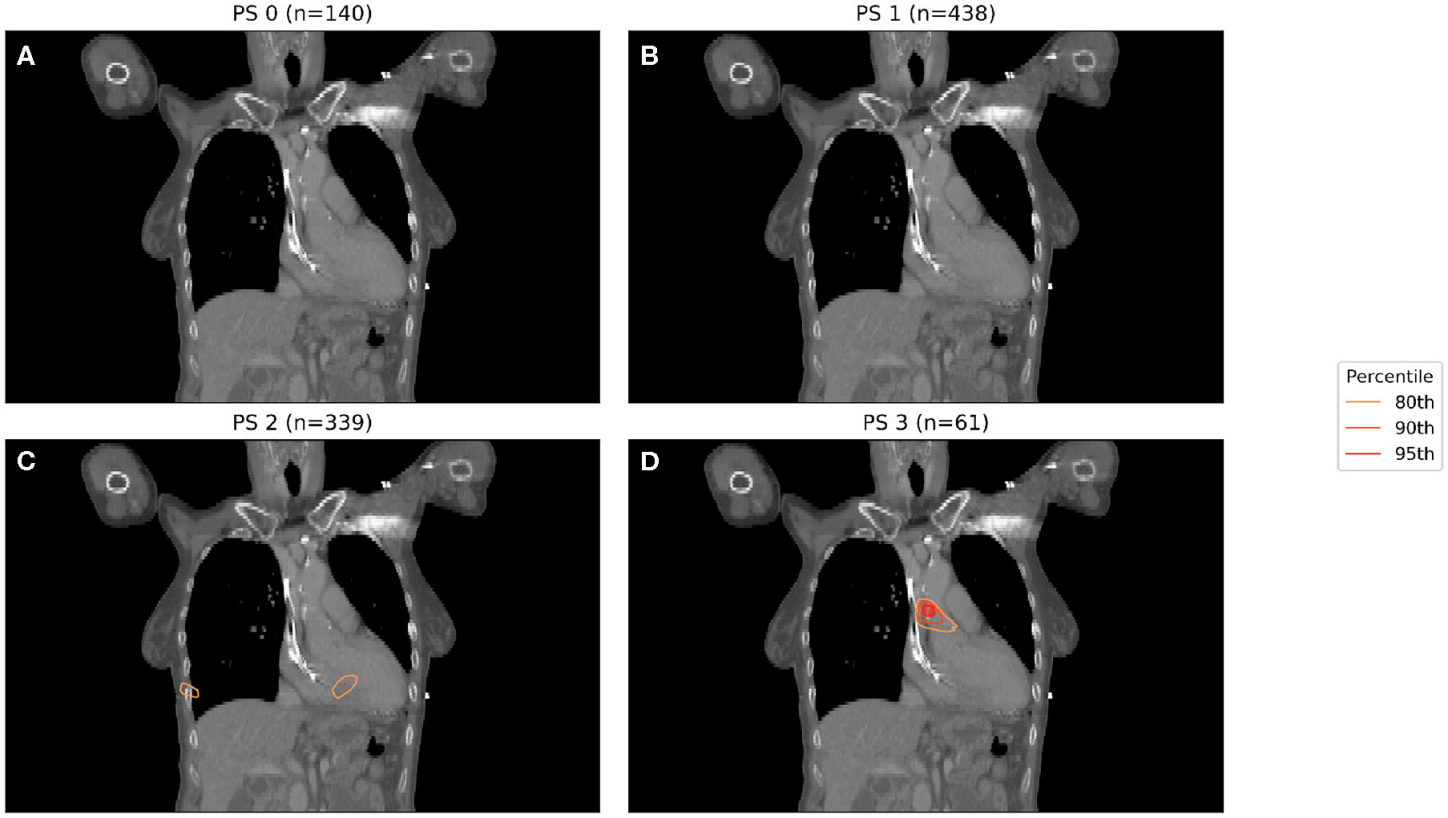
IBDM repeated for each of the four performance statuses considered (0 in **A**, 1 in **B**, 2 in **C**, 3 in **D**). One patient with PS 4 was omitted. Percentile refers to the percentile of the t distribution approximated by permutation testing used to assess significance, i.e., 95th percentile corresponds to *p* = 0.05. Percentile refers to the percentile of the t distribution approximated by permutation testing used to assess significance, i.e., 95th percentile corresponds to *p* = 0.05, all analyses used 1,000 permutations. Statistically significant regions (regions inside the 95th percentile; *p* ≤ 0.05) after permutation testing where dose differs between living and dead patients are indicated by contours in red, while less significant regions are shown in other colors. No anatomically plausible, statistically significant region where dose differs between living and dead patients was found, except for the PS three patients **(D)**.

In Figure 1, binary IBDM has been performed in the four sub-cohorts defined by splitting on PS. The region highlighted in red is statistically significant with *p* = 0.05. In these significant regions, an excess of dose is associated with decreased overall survival at 12 months post-RT.

A significant region in Figure 1 only exists for those patients whose PS is 3, with no statistically significant region being found for patients with lower PS. The region highlighted in Figure 1 is consistent with that previously identified in this cohort, being located in the base of the heart, and in a similar anatomical location to the most significant region in the previous analysis (11). In this analysis, the majority of patients had PS 1 (438 patients, 355 events) or PS 2 (339 patients, 276 events), with a small number being PS 0 (140 patients, 104 events) or 3 (61 patients, 52 events).

**Figure 2:**
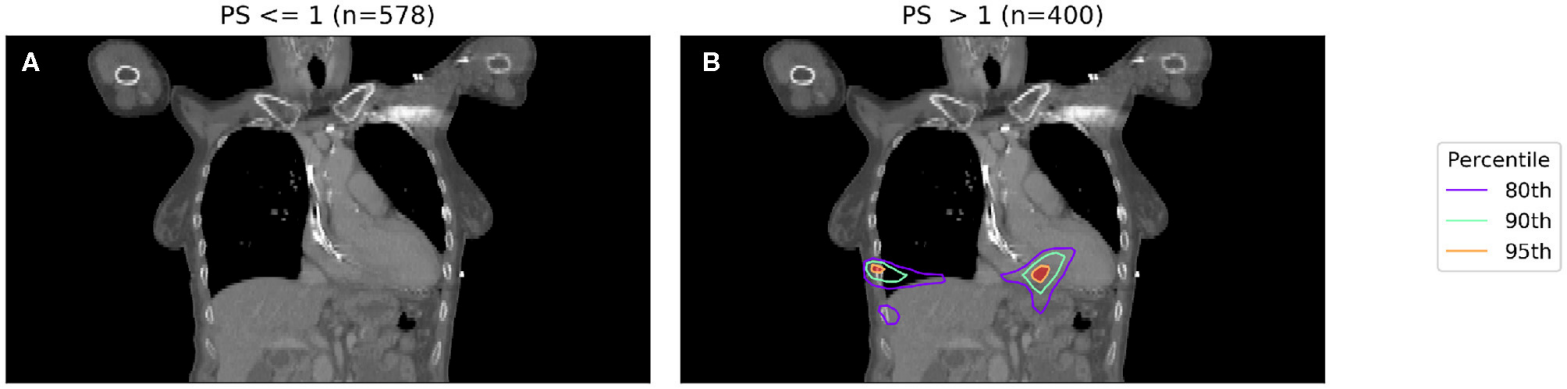
IBDM repeated for low (PS 0-1, shown in **A**) and high (PS 2-3, shown in **B**) PS patients. Percentile refers to the percentile of the t distribution approximated by permutation testing used to assess significance, i.e., 95th percentile corresponds to *p* = 0.05, all analyses used 1,000 permutations. In the low PS patients, no significant regions (*p* = 0.05 in both) were identified (global *p* = 0.19), whereas in the high PS patients a region is identified in the body of the heart (global *p* = 0.001). The region identified in high PS patients is incompatible with prior results at this significance level reported by McWilliam et al. (11).

To account for the inaccuracy of PS, and to obtain larger and better balanced cohorts, we dichotomize PS into those patients with PS < 1 and those with PS ≥ 1. The results of two binary IBDM analyses in cohorts split based on dichotomized PS is shown in Figure 2.

In Figure 2, a significant region is identified for those patients with poorer performance, however in this case it is not compatible with results found in a previous analysis of this cohort (11), which identified the base of the heart as a dose sensitive structure. The location of the significant region, around the inferior edge of the lungs, may actually be indicating the effect of tumor size, with larger tumors having larger high dose regions—this leads to a spuriously significant region in which dose difference and survival are both driven by tumor size, which is un-accounted for. Naively, one may now try to account for tumor size by splitting each sub-cohort (already split according to PS) further according to their tumor size.

### 3.2. Per-voxel Cox IBDM

**Figure 3:**
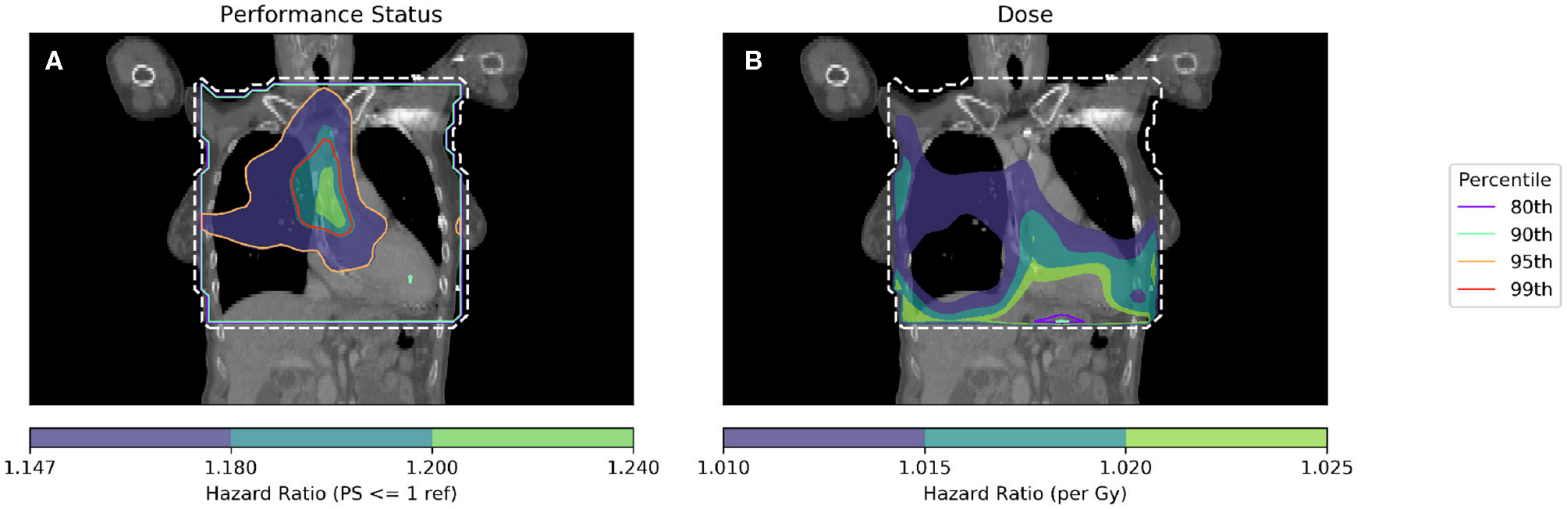
Cox-IBDM performed considering only PS **(A)** and dose **(B)**. Shown by the dashed white line is the region inside the mask, where hazard ratio calculations were performed. In unfilled contours, hazard ratio percentiles are shown, while hazard ratios are shown in filled contours. Percentile refers to the percentile of the hazard ratio distribution approximated by permutation testing used to assess significance, i.e., 95th percentile corresponds to *p* = 0.05, all analyses used 1,000 permutations. Note that the hazard ratio scale is different in each subfigure. For the PS hazard map **(A)**, all regions with HR > 1.147 are statistically significant (*p* < 0.05); a region in the base of the heart is identified with a significant (when permuting the dose) hazard ratio of around 1.2, taking account of the effect of dose. In the dose hazard map **(B)**, no region is statistically significant when permuting the dose; hazard ratios shown are for illustration, having *p* > 0.1.

Hazard ratios were produced and are shown in Figure 3. The further usefulness of the method is demonstrated by including additional clinical variables: age and the cube root of the tumor size, both of which are important predictive clinical factors (6, 23). In Figure 3, a Cox regression is performed per-voxel taking account of dichotomized PS as in Figure 2, but also dose in each voxel. This technique allows analysis of the dose sensitivity of the entire cohort while controlling for PS. The equation of the Cox regression used in Figure 3 is shown (Equation 1):

(1) $$\begin{array}{l} \ln\left(\lambda_{p,i}\left(t\right)|X_{p,i}\right)=\ln\left(\lambda_{0,p}\left(t\right)\right)+\beta_{PS>1,i}X_{PS>1,p}+\beta_{dose,i}X_{dose,p,i} \end{array}$$

where λ(*t*) is hazard function estimated from the data *X*, and λ_0_(*t*) is the time-dependant baseline hazard function. *X*_*PS*>1_ is a dichotomous variable whose value is 1 when a patient's performance status is >1. Beta is the Cox model coefficient for dichotomized PS or dose respectively. Subscript p refers to patients, while subscript i refers to dose distribution voxels. Dose in each voxel is a continuous variable.

By combining spatially invariant clinical data with the spatially varying dose data, it is possible to identify regions in which clinical variables appear to have a stronger effect. In Figure 3 this effect is seen for PS > 1, where those patients with higher PS have worse overall survival linked to dose in a region of the base of the heart. The implication of this analysis is that patients with poor performance status (i.e., general fitness/health/indication of co-morbidities) are less tolerant of dose in the base of the heart.

**Figure 4:**
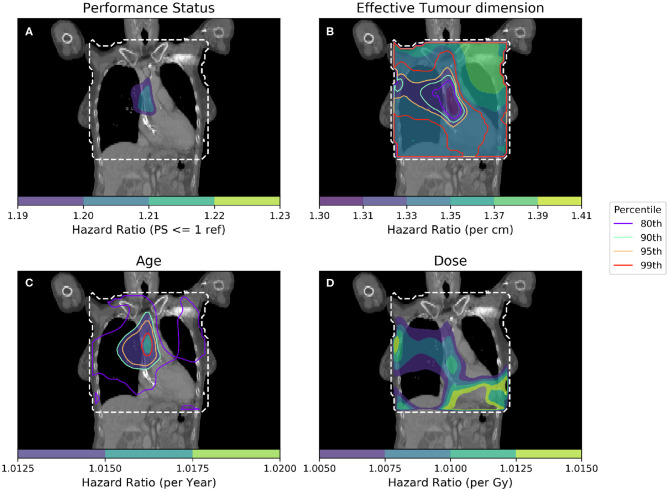
Cox-IBDM taking account of the most significant clinical variables [binary PS **(A)**, Tumor size **(B)**, Age **(C)**], and dose **(D)**. Again, the mask used is indicated by the white dashed line, significance indicated by the unfilled contours and hazard ratio by the filled contours. Percentile refers to the percentile of the hazard ratio distribution approximated by permutation testing of the summary statistic used to assess significance, i.e., 95th percentile corresponds to *p* = 0.05, all analyses used 1,000 permutations. Note that hazard ratio scales are different in each sub-figure, and that the hazard ratio for tumor size and age are both highly significant, regardless of dose.

To further isolate the relationship between clinical variables and dose, it is necessary to control for several more clinical variables when performing analyses. In Figure 4, IBDM is performed using a per-voxel Cox regression controlling for the most significant clinical variables as identified in other studies (11): effective tumor dimension, age and PS. In Figure 4, the equation used is

(2) $$\begin{array}{r} \ln\left(\lambda_{p}\left(t\right)|X_{p,i}\right)=\ln\left(\lambda_{0,p}\left(t\right)\right)+\beta_{PS>1,i}X_{PS>1,p}+\beta_{ETD,i}X_{ETD,p}\\ +\beta_{Age,i}X_{Age,p}+\beta_{dose,i}X_{dose,p,i} \end{array}$$

where λ(*t*) is the hazard function estimated from the data *X*, and λ_0_(*t*) is the time-dependant baseline hazard function. Again, *X*_*PS*>1_ is a dichotomous variable whose value is 1 when a patient's performance status is >1. β_*PS*_ is the Cox model coefficient for dichotomized PS, β_*ETD*_ is the Cox model coefficient for effective tumor dimension, β_*age*_ refers to the Cox model coefficient for age and β_*dose*_ is the Cox model coefficient for dose. Subscript p refers to patients, while subscript i refers to dose distribution voxels. Effective Tumor Dimension is abbreviated ETD. Age, ETD, and dose are treated as continuous variables.

As in Figure 3, an important region is highlighted in the base of the heart where dose has a larger detrimental effect on those patients whose PS is poor. However, when considering four covariates this region is no longer statistically significant. Instead, an overlapping region in which the patient's age is important becomes significant, indicating the interplay between covariates across the anatomy. The hazard ratio for effective tumor dimension is largely spatially invariant, and is significant in all voxels tested, except the base of the heart where other covariates appear to be more important. A reduction in tumor dimension hazard in the region of the base of the heart may indicate that dose to the base of the heart is a competing effect, alongside tumor size. However, this reduction is mirrored by an increase in the hazard ratio associated with dose, likely because larger tumors lead to larger doses across the mediastinum. All covariates were statistically significant (*p* = 0.05) when permuting dose, apart from the dose and PS. In this analysis, 939 patients were included, the number again reduced relative to the previous analysis due to missing data. This is a proof of principle analysis using some common clinical variables and illustrating the hazard ratio maps resulting from the method; in principle there is no limit to the number of clinical variables that could be included.

### 3.3. The Effect of GTV Dose

**Figure 5:**
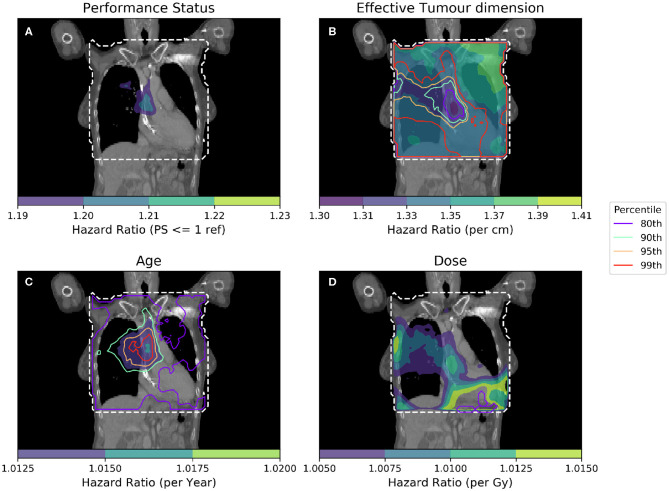
Cox-IBDM taking account of the most significant clinical variables [dichotomized PS **(A)**, Tumor size **(B)**, Age **(C)**], and dose **(D)**. Again, the mask used is indicated by the white dashed line, significance indicated by the unfilled contours and hazard ratio by the filled contours. Percentile refers to the percentile of the hazard ratio distribution approximated by permutation testing of the summary statistic used to assess significance, i.e., 95th percentile corresponds to *p* = 0.05, all analyses used 1,000 permutations. In this analysis, dose in voxels within the GTV is treated like missing data, meaning that for the regression in voxels labeled as GTV for a particular patient, that patient is excluded from the analysis.

In Figure 5 the hazard ratios and significance thresholds are shown for a per-voxel Cox regression in which GTV voxels are treated as missing data.

The overall indication from this analysis is the same. The shape and location of statistically significant regions does not change very much, but the iso-significance contours become much more noisy; this implies that the permutation test methodology is robust to variable patient numbers across the anatomy, but that more patients may be required when excluding the GTV region from the analysis.

## 4. Discussion

In this work, we aim to address one of the remaining inadequacies of standard image based data mining—the lack of consideration for clinical covariates during region of interest identification. This work has performed, for the first time, survival analysis per-voxel across a large patient population, producing hazard ratio maps showing that clinical variables have a spatially varying hazard when dose across anatomy is taken into account. The power of the per-voxel Cox method, over binary IBDM, is in the ability to use the full cohort: in this analysis data from 939 patients was used. This information has never been shown before, as standard Cox-regression analysis can only handle 0-dimensional parameters (for instance DVH parameters), where spatial information is lost. The approach developed in this work allows greater understanding of the variation in risk across patient anatomy and offers further opportunities to better create the optimal treatment plan for radiotherapy patients.

Using binary IBDM and splitting our cohort based on patients' performance status, we have identified a sub-population of patients whose PS is poor in whom dose to the base of the heart is strongly associated with poorer survival, however this region was not identified in patients whose PS is better. Since PS is a subjective measure, grouping patients on raw PS score is ill-advised; however, a different statistically significant region was found for patients grouped PS ≤ 1 and PS > 1. The variability in regions identified is strongly dependent on the grouping of the data, with competing effects, and small unbalanced cohorts contributing to a reduction in analysis power. No anatomically plausible region is identified when accounting for the inaccuracy of PS as a measure of patient health by splitting the cohort based on PS ≤ 1 and PS > 1, illustrating the inappropriateness of dividing a cohort based on a covariate as a way to control for that covariate.

Per-voxel IBDM using a Cox regression to account for confounding clinical variables offers an alternative method to perform IBDM while accounting for clinical factors and retaining a large cohort. The method highlights the interplay of dose with clinical variables, and has identified a region in the heart where patients with poorer PS (*PS*>1) who receive excess dose have worse survival. Care must be taken in the interpretation of hazard ratio maps produced using this method, as there is considerable scope for interactions between covariates and the addition of spatial variation can make interpretation even more challenging. An interesting extension of the Cox PH model would be the inclusion of interaction terms between variables such as PS and dose; however, the interpretation of such an analysis is difficult and is beyond the scope of this current work. The observed asymmetry in the hazard ratios shown in Figures 3, 4 suggests there may be some effect due to tumor location. We have investigated the location of tumors in this cohort previously (24), and while there is a difference in survival between left- and right-sided tumors (25), there is no spatial correspondence between the likely tumor location and the patterns seen in the hazard ratio maps. However, given that our analysis identifies regions in the heart where dose is detrimental, it would be interesting to include the location of the tumor, relative to the heart, in future analyses.

In IBDM, since all patients must be non-rigidly registered to a single reference anatomy for the analysis, the subsequent analysis is subject to uncertainty due to the registration process, and may be subject to bias due to the selection of reference patient. We have presented our methodology for assessing the registration accuracy in this cohort, which involves analysis of heart segmentations propagated subset of our patients to the reference. This allows us to say with some certainty that our registration accuracy is acceptable within the heart, but does not offer insight into the registration quality within the lungs. The choice of reference patient will obviously impact the registration, and therefore influence the dose distributions in the common frame of reference. We have previously investigated the impact of reference patient selection on the result of IBDM and found only small differences (26), in spite of large variations in reference anatomy.

It may be argued that dose to the GTV is intended to be curative, whereas dose to normal tissue is not, and therefore the two cannot be analyzed in the same way. We have investigated the effect of removing the dose in the GTV. In summary the overall conclusion of the analysis is unchanged, but the effect of removing the GTV dose on the permutation test output is to introduce noise. The changes in the permutation test are due to the summary statistic used (most extreme hazard), and the increased size of confidence intervals where GTV dose has been excluded. We believe that keeping the dose to the GTV in the analysis offers the cleaner and more robust analysis, and allows for situations in which there is no GTV, only a CTV which may include normal tissue. If the GTV is to be excluded, we recommend varying the number of patients analyzed per-voxel as this is the more statistically robust and extensible method.

By treating GTV voxels as missing data, we took the simplest possible approach and excluded those patients from the regression in GTV voxels. This approach is valid provided voxels are missing at random, and that less than 5% of patients are excluded in any given voxel (27). The largest number of patients excluded in any voxel of this analysis is 56 (6%) and, at a per-voxel level, exclusion is effectively random as the location of the GTV is random. It may be possible to use more sophisticated techniques for dealing with missing data, such as multiple imputation. However, given that at most 6% of patients were excluded in a single voxel, simply using complete case analysis is sufficient for this analysis; in analyses where many GTVs overlap in the reference anatomy, this will not be the case (28) and care must be taken to ensure that the missingness is not informative.

A weakness in all studies on retrospective data is poor outcome data, including actual cause of death. The analysis here has been performed using overall survival as an endpoint, but since the cause of death remains unknown it is impossible to properly disentangle the effect of dose in the base of heart from other factors. We hypothesize that dose to the base of the heart is predictive of survival. While this hypothesis makes instinctive sense, it is necessary to consider that the cause of death for these patients is unknown. The statistically significant region identified using IBDM extends well into the right lung, implying that there may be more than one effect at play. For example, Radiation Induced Lung Dysfunction (RILD) has been demonstrated in rat models, with resultant vascular remodeling leading to pulmonary hypertension (29). RILD has also been identified in retrospective analysis of data from lymphoma patients (12), though these patients have very different comorbidities to the group analyzed in this work. It is also true that the right lung is larger than the left, and therefore conceivable that dose delivered in the right lung may impact survival and present a competing effect alongside cardiac dose that this study is at present unable to differentiate (7). Were data available, Cox-IBDM could be used to perform a time-to-event analysis for cardiac events which should further clarify the relative importance of dose sensitive regions. However, given that such datasets are likely to be small, or have a small event rate it will be necessary to carefully investigate the exclusion of dose to the GTV; while it may influence the outcome, it will very likely dominate other regions in the permutation test due to the changes to confidence intervals.

Similarly, the lack of high-quality comorbidity data is a limitation in this analysis as in a previous analysis of this cohort. Here however, available co-morbidity data can be incorporated directly into the IBDM process, meaning important dose-sensitive regions may be localized that depend on the fitness of the patient as well as on the characteristics of their disease, including histology. By accounting for comorbidities in the generation of dose-sensitive ROIs the ROIs should be more robust, making testing hypotheses generated by IBDM more straightforward. In this analysis we have used performance status as a crude but widely available surrogate for comorbidities, and identified those patients whose performance is worst as being at the greatest excess risk during radiotherapy; this hypothesis could be readily tested by imposing a stricter limit on heart dose for those patients whose performance is poor. With more detailed comorbidity data, this hypothesis could be further refined.

This study has evaluated dose effect on overall survival after radiotherapy, either with a dichotomized analysis between patients surviving up to 12 months, or with a per voxel survival analysis. While the methods applied are state-of-the-art, the clinical endpoint is important yet limited. There are numerous confounding factors which have an effect on overall survival and some of them will not be captured in the data we have analyzed. A more compelling analysis would use, for example, cardiac death after radiotherapy; however, this data is difficult to produce in sufficient quantities to perform IBDM. More refined endpoints will be needed to refine IBDM generated hypotheses, but hypotheses generated with an unequivocal but limited endpoint are highly important when designing future studies.

In binary IBDM, statistical significance is assessed by permuting labels to dose data (e.g., dead/alive) and re-calculating the test statistic; equivalently, it would be possible to permute the dose distributions with respect to the labels, but this is not done as it is computationally expensive. In this work, each dose distribution is associated with several variables, including the time to event and status label but also any covariate information to be used in the Cox regression. To produce permuted regressions here, we have permuted entire collections of covariate and event data with respect to the dose. This is identical to permuting the dose distributions, but more computationally efficient. As in our binary analysis, the most extreme value in each channel is recorded for each permuted analysis, and the 95th percentile of the permuted distribution used to give a significance threshold for that variable. Due to the nature of the analysis, assessing significance in this way is susceptible to errors where the regression does not converge (e.g., areas with very low dose). It is possible to exclude these regions by requiring a minimum mean dose threshold for a voxel to be included in the analysis, though this may cause important regions whose mean dose is less than the threshold to be ignored. Threshold free cluster enhancement (TFCE) (30) has been used in other analyses and we are exploring its application in this type of analysis.

In this work, we have performed permutation testing to assess statistical significance using the max-T method proposed by Chen et al. (9). In similar work by Sörensen et al. (17), a false discovery rate (FDR) approach was used to adjust analytical p-values derived from the Cox model fitting. While this may be appropriate in that analysis, we feel that the use of a full permutation is preferable in our analysis, due to the presence of strong correlation between neighboring voxels in a dose distribution, which must be carefully taken into account in FDR. In the analysis by ten Kate et al. (16), permutation testing was used, but every voxel was treated independently in the construction of the permutation distribution. Again, this approach is inappropriate for our data due to the presence of correlations in the dose distribution. Our approach calculates permutations in which the dose is effectively associated with a different patient in every permutation, which removes the impact of correlations between voxels in the dose distribution for the purposes of permutation testing.

In summary, IBDM using a per-voxel Cox regression has, for the first time, produced three-dimensional maps of hazard for clinical variables when considering radiotherapy dose. These hazard ratio maps have been used to identify dose-sensitive regions compatible with those seen using a standard IBDM technique, and highlight the interaction between dose and other clinical variables such as PS. By using a Cox-based IBDM approach, it is possible to do time-to-event studies including clinical variables that will further clarify the relative importance of dose-sensitive structures, making the clinical hypotheses generated using IBDM more easily testable and robust.

## Data Availability Statement

The datasets presented in this article are not readily available because ethical permission was not granted for general publication. Requests to access the datasets should be directed to Prof. Marcel van Herk (marcel.vanherk@manchester.ac.uk).

## Ethics Statement

Our retrospective analysis of routine radiotherapy data was approved by the local institutional information governance and ethics committee (The Christie NHS Foundation Trust and Caldicott Committee). All data was anonymized, and the work carried out according to a protocol approved by the Caldicott committee.

## Author Contributions

AG implemented code and performed the primary analysis. EV performed non-rigid registration and registration inaccuracy analysis. AM collected data and provided additional analysis. MA provided additional analysis. MH conceived the experiment. All authors reviewed the manuscript.

## Conflict of Interest

The authors declare that the research was conducted in the absence of any commercial or financial relationships that could be construed as a potential conflict of interest.
